# Charting clonal evolution and behavior with GoT-Multi

**DOI:** 10.1016/j.xgen.2025.101077

**Published:** 2026-01-14

**Authors:** Jonas A. Gudera, Vijay G. Sankaran

**Affiliations:** 1 Division of Hematology/Oncology, Boston Children’s Hospital, Harvard Medical School, Boston, MA, USA; 2 Department of Pediatric Oncology, Dana-Farber Cancer Institute, Harvard Medical School, Boston, MA, USA; 3 Howard Hughes Medical Institute, Boston, MA, USA; 4 Broad Institute of MIT and Harvard, Cambridge, MA, USA; 5 Harvard Stem Cell Institute, Cambridge, MA, USA

## Abstract

Tracking clonal evolution is critical to fully define the mechanisms of normal physiology and the disruptions of these processes in disease. In this issue of *Cell Genomics*, Pak and Saurty-Seerunghen et al. describe the development of Genotyping of Transcriptomes for multiple targets and sample types (GoT-Multi) and show how this new technology enables insights into cellular states that mediate clonal evolution in diseases, such as the Richter transformation of chronic lymphocytic leukemia, while also revealing convergence of cell states, even with distinct driver mutations.

## Main text

**Figure 1:**
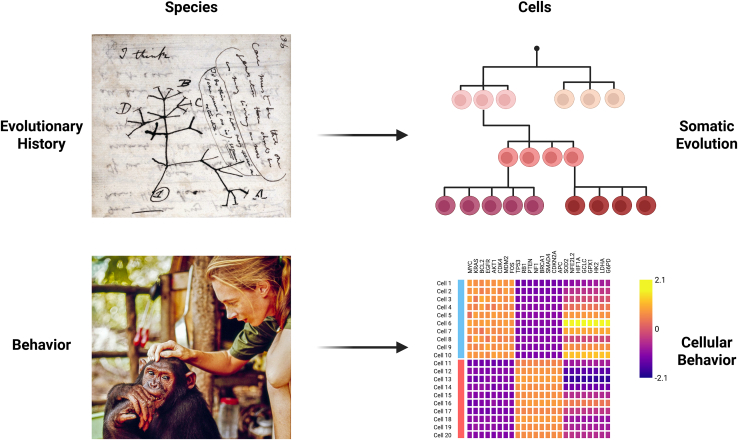
Hallmarks of studying evolution and somatic evolution Charles Darwin’s notebook in 1837 depicting his first sketch of an evolutionary tree (top left; credit: transmutation of species [1837–1838] Darwin Online via Wikipedia), Jane Goodall with a chimpanzee (bottom left; credit: Getty Images), schematic lineage tree of cells (top right; created with BioRender), and schematic gene expression heatmap (bottom left; created with BioRender).

Evolutionary biology has reshaped how we understand life. Since Darwin formalized the idea of common descent (Figure 1), the rise of genomic technologies has refined our understanding of evolutionary relationships among species. Closely related species that share the overwhelming majority of their DNA can exhibit strikingly different phenotypes and behaviors, as exemplified by humans and closely related primates. Thus, genetic information alone is incapable of predicting the full spectrum of phenotypes and behaviors we would anticipate for a species. To properly achieve this level of understanding, detailed studies of species in their natural environments are needed, as nicely demonstrated by the late Jane Goodall’s extensive work on wild chimpanzees (Figure 1).

Species evolve over time through the accumulation of mutations and selective pressures. A similar logic applies when studying the multitude of cells within an individual. Somatic evolution refers to the lifelong accumulation of genetic alterations within somatic cells and to the influence of these changes on the relative fitness and behavior of these individual cells.^1^ Jointly resolving somatic evolution and cellular behavior at single-cell resolution has been a long-standing technical challenge due to data sparsity, noise, and sequencing costs. Nonetheless, methods that couple cell-state profiling with nuclear variant detection, including Seq-well, Genotyping of Transcriptomes (GoT), and Nanoranger, have begun to bridge this gap by linking genotype to transcriptomic phenotype at scale.^2^^,^^3^^,^^4^

In this issue, Pak and Saurty-Seerunghen et al. introduce a novel method termed GoT for multiple targets and sample types (GoT-Multi) providing previously unappreciated biological insights into the phenomenon of Richter transformation (RT) that rarely occurs in individuals with chronic lymphocytic leukemia (CLL).^5^

GoT-Multi introduces a probe-based strategy for high-throughput mRNA quantification that is compatible with cell hashing, enabling multiplexing of multiple samples within a single experiment. Unlike the original GoT method, which relied on targeted complementary DNA (cDNA) amplification, GoT-Multi uses hybridization with pre-designed probe pools for capture-based single-cell genotyping. For each variant of interest, genotyping probes must be designed in advance to include both wild-type and mutant alleles. Each probe carries a unique barcode corresponding to its allelic status and to the specific locus under study, enabling a two-tiered validation of variant calls: one through the probe sequence itself and another through the probe barcode. Following probe hybridization, cells are prepared for gel bead-in-emulsion formation, followed by gene expression-, hashtag oligo-, and genotyping library construction. Importantly, this protocol can be broadly applied to a variety of sample types, including formalin-fixed paraffin-embedded (FFPE) tissue or fresh-frozen samples, that are often obtained in clinical settings.

Sequencing data are refined using a computational pipeline that applies machine learning (ML)-based denoising, GoT-Multi ML. The framework models nonlinear relationships between assay features, such as probe GC (guanine and cytosine) content, read depth, and background signal, to distinguish true variant-harboring cells from artifacts caused by nonspecific hybridization, ambient RNA contamination, or PCR recombination. Using admixed reference populations, the authors demonstrate that inclusion of wild-type probes reduces false-positive rates by up to 50%, and ML-based denoising achieves an overall error reduction of up to 88%, which is validated in controlled mixing experiments.

Together, these innovations yield three major improvements over prior technologies. First, GoT-Multi works on FFPE tissues, overcoming a major limitation of previous single-cell genotyping methods. Second, it overcomes poly-A biases, allows profiling of more mutations, and supports multiplexed experiments that generate internal controls. Third, its integrated ML framework allows denoising of the genotyping data.

Pak and Saurty-Seerunghen et al. first validate GoT-Multi (with the ML framework) in a cell-line mixing experiment using two breast cancer cell lines, evaluating the genotyping rate (fraction of cells with an assigned genotype) and genotype accuracy. To demonstrate the generalizability of their denoising approach, they further apply the GoT-Multi ML framework to an independent GoT dataset, in which there is also improved variant calling.

The authors deploy GoT-Multi to study RT—the evolution of CLL, a low-grade malignancy of mature B cells, into aggressive large B cell lymphoma (LBCL). RT arises in a minority of CLL patients, often after therapy, and carries a poor prognosis, with resistant subclones frequently harboring *BTK* or *PLCG2* mutations. Across five patients, fluorescence-activated cell sorting was used to partition CLL, LBCL, T cells, and residual compartments, followed by GoT-Multi profiling of 18 somatic mutations. Variants mapped to distinct transcriptional states; for example, mutations in *B2M*, *IRF8*, *JUNB*, and *EMD* show enrichment in LBCL-associated states, whereas other subclonal mutations (e.g., *SRRM2*) appear decoupled from malignant phenotypes. Where available, integration with bulk DNA and copy-number data further refined subclonal architecture, exemplified by one case showing coherent losses on chromosomes 4, 15, and 17 associated with *POU2F2*, *B2M*, and *EMD* mutations.

This approach also provided some surprising findings in clonal evolution. For example, in one patient, three distinct *PLCG2* mutations were identified, two of which were known therapy-resistant variants. These resistant alleles mapped to inflammatory and immune-response cell states, whereas the non-resistant mutation localized to a proliferative cell state. Similarly, a *BTK* mutation associated with treatment resistance was enriched in inflammatory and immune-response cell states. The analysis further revealed the variability of somatic mutation effects as a function of the underlying cell state; for example, the *PLCG2* p.S1079R clone displayed distinct sets of dysregulated genes depending on whether cells were in immune-activated or proliferative states.

Therapy-resistant subclones carrying *PLCG2* or *BTK* mutations displayed upregulation of tumor necrosis factor (TNF) and interferon signaling pathways together with suppression of cell-cycle genes. Other subclones bearing *SRRM2* (p.T2409K) or *JUNB* (p.E6Ter) mutations also activated TNF signaling, suggesting that genetically distinct clones can converge on shared inflammatory transcriptional programs that may underlie therapy resistance in some cases.

Beyond inflammation, subclones carrying mutations in *IRF8* or *POU2F2*, both of which encode key B cell transcription factors, were linked to enhanced MYC activity, connecting subclonal genotypes to the well-known MYC activation signature in RT. Together, these findings reinforce a model in which diverse subclonal genotypes drive changes in cell state that converge on shared oncogenic pathways, a finding only made possible by linking specific genotypes with insights on cellular behavior.

Having established performance and biological readouts in fresh-frozen samples, the authors next assessed GoT-Multi performance on archival FFPE samples. In an RT case, the method generated high-quality single-cell transcriptomes with lower, albeit interpretable, genotyping rates across nine mutations, compared with fresh-frozen samples (up to 29% vs. up to 80%). Clonal reconstruction revealed a branched hierarchy in which an *FBXW7*-mutated subclone later acquired *NOTCH1* mutations, displaying transcriptional patterns similar to therapy-resistant *PLCG2* and *BTK* clones.

Overall, GoT-Multi demonstrates high sensitivity and specificity across fresh-frozen and FFPE samples, enabling joint profiling of genotype and cell state at single-cell resolution. However, genotyping efficiency still depends on the targeted locus, expression level, and probe design, which may constrain certain applications.

Because GoT-Multi requires prior knowledge of mutated alleles, it is best suited for malignancies where clonal variants can be reliably detected from bulk sequencing. At the same time, coupling this clonal resolution with additional markers that can track subclonal evolution, such as emerging approaches to detect naturally occurring barcodes derived from distinct nuclear mutation classes, mitochondrial variants, or epimutations could further resolve subclonal diversity within a given driver-mutation-defined clone.^6^^,^^7^^,^^8^^,^^9^ Indeed, combining assessment of mitochondrial mutations with driver mutations has been valuable for improving clonal resolution in a method combining GoT with chromatin accessibility assessments.^10^ Given advances in single cell detection of such variation, it might be possible that these additional barcodes for tracking subclonal dynamics could be incorporated into a workflow adapted from GoT-Multi in the future.

By linking somatic genotyping with transcriptomic state, GoT-Multi illuminates evolutionary dynamics within tumors, revealing both convergent processes (e.g., shared MYC activation and TNF signaling across subclones) and divergent ones (context-dependent mutation effects). In doing so, it exemplifies how integrating genetic and cellular behavior information at the single-cell level can uncover new principles of cancer evolution and potential therapeutic vulnerabilities.

## Acknowledgments

We are grateful to members of the Sankaran laboratory for feedback. J.A.G. is supported by a Boehringer-Ingelheim MD Fellowship. The laboratory of V.G.S. is supported by the Howard Hughes Medical Institute, the Alex’s Lemonade Stand Foundation, the Mathers Foundation, the Edward P. Evans Foundation, the Gates Foundation, Blood Cancer United, Care for Rare America, and the National Institutes of Health (NIH; R01CA265726, R01CA292941, R33CA278393, R01DK103794, and R01HL146500). V.G.S. is an investigator of the Howard Hughes Medical Institute.

## Declaration of interests

V.G.S. serves as an advisor to Ensoma, Cellarity, and Beam Therapeutics, all unrelated to this work.
